# Results of Adjustable Trans-Obturator Male System in Patients with Prostate Cancer Treated with Prostatectomy and Radiotherapy: A Multicenter Study

**DOI:** 10.3390/jcm12144721

**Published:** 2023-07-17

**Authors:** Javier C. Angulo, Carlos Téllez, Alessandro Giammò, Carmen González-Enguita, Sandra Schoenburg, Fabian Queissert, Juliusz Szczesniewski, Raquel González, Antonio Romero, Andreas Gonsior, Francisco E. Martins, Tiago Antunes-Lopes, Francisco Cruz, Keith Rourke

**Affiliations:** 1 Clinical Department, Faculty of Biomedical Science, Universidad Europea, Carretera de Toledo, Km 12.500, Getafe, 28905 Madrid, Spain; soyctf@hotmail.com; 2 Department of Urology, Hospital Universitario de Getafe, Carretera de Toledo, Km 12.500, Getafe, 28905 Madrid, Spain; juliusz.szcz@gmail.com; 3 Department of Neuro-Urology, CTO/Spinal Cord Unit, AOU Città Della Salute e Della Scienza di Torino, Via Zuretti 24, 10126 Torino, Italy; giammo.alessandro@gmail.com; 4 Department of Urology, Hospital Fundación Jiménez Díaz, Av. de Los Reyes Católicos, 2, 28040 Madrid, Spain; cgenguita@fjd.es (C.G.-E.); rgonzalezl@fjd.es (R.G.); 5 Department of Urology and Kidney Transplantation, Martin Luther University, Ernst-Grube-Straße 40, 06120 Halle (Saale), Germany; sandra.schoenburg@uk-halle.de; 6 Department of Urology and Pediatric Urology, University Hospital Muenster, Albert-Schweitzer-Campus 1, 48149 Münster, Germany; fabian.queissert@ukmuenster.de; 7 Department of Urology, Hospital Universitario Morales Meseguer, Avd. Marqués de Los Vélez s/n., 30008 Murcia, Spain; antonio.romero6@carm.es; 8 Klinik und Poliklinik für Urologie, University of Leipzig, Liebigstraße 20, 04103 Leipzig, Germany; andreas.gonsior@medizin.uni-leipzig.de; 9 Department of Urology, Centro Hospitalar Universitário de Lisboa Norte, Hospital Santa María, Av. Prof. Egas Moniz MB, 1649-028 Lisboa, Portugal; faemartins@gmail.com; 10 Department of Urology, Centro Hospitalar São João and Faculty of Medicine of University Porto, Alameda Prof. Hernâni Monteiro, 4200-319 Porto, Portugal; tiagoantuneslopes@gmail.com (T.A.-L.); cruzfmjr@med.up.pt (F.C.); 11 I3S Institute, R. Alfredo Allen 208, 4200-135 Porto, Portugal; 12 Department of Urology, Alberta University, Hospital Edmonton, 8440 112 St. NW, Edmonton, AB T6G 2B7, Canada; krourke@ualberta.ca

**Keywords:** adjustable trans-obturator male system, post-prostatectomy incontinence, adjuvant radiotherapy, outcomes, satisfaction

## Abstract

(1) Background: Treatment of male stress incontinence in patients with prostate cancer treated with radical prostatectomy and adjuvant pelvic radiation is a therapeutic challenge. The efficacy and safety of the adjustable trans-obturator male system (ATOMS) in these patients is not well established, despite the general belief that outcomes are worse than in patients without radiation. (2) Methods: Retrospective multicenter study evaluating patients treated with silicone-covered scrotal port (SSP) ATOMS implant after radical prostatectomy and radiotherapy in nine different institutions between 2016 and 2022. The primary endpoint was dry patient rate, defined as pad-test ≤ 20 mL/day. The secondary endpoints were complication rate (defined using Clavien–Dindo classification), device removal and self-perceived satisfaction using the Patient Global Impression of Improvement (PGI-I) scale. Wilcoxon rank-sum test, Fisher’s exact test and logistic regression were performed using stepwise method with a 0.15 entry and 0.1 stay criteria. (3) Results: 223 patients fulfilled the criteria for inclusion and 12 (5.4%) received salvage prostatectomy after radiation and 27 (12.1%) previous devices for stress incontinence. After ATOMS adjustment, 95 patients (42.6%) were dry and 36 (16.1%) had complications of any grade (grade I, *n* = 20; grade II, *n* = 11; grade III, *n* = 5) during the first 3 months postoperatively. At a mean of 36 ± 21 months follow-up, the device was explanted in 26 (11.7%) patients. Regarding self-perceived satisfaction with the implant, 105 of 125 patients (84%) considered themselves satisfied (PGI-I 1 to 3). In the univariate analysis, dryness was associated to younger age (*p* = 0.06), primary prostatectomy (*p* = 0.08), no previous incontinence surgery (*p* = 0.02), absence of overactive bladder symptoms (*p* = 0.04), absence of bladder neck stricture (*p* = 0.001), no need of surgical revision (*p* = 0.008) and lower baseline incontinence severity (*p* = 0.0003). Multivariate analysis identified absence of surgical revision (*p* = 0.018), absence of bladder neck stricture (*p* = 0.05), primary prostatectomy (*p* = 0.07) and lower baseline incontinence severity (*p* < 0.0001) were independent predictors of dryness. A logistic regression model was proposed and internally validated. (4) Conclusions: ATOMS is an efficacious and safe alternative to treat male incontinence after radical prostatectomy and adjuvant radiotherapy. Factors predictive of dryness are identified in this complex scenario to allow for better patient selection.

## 1. Introduction

Radical prostatectomy has been the most commonly used treatment option for patients with intermediate and high-risk clinically localized prostate cancer [1]. However, despite the refined surgical technique and patient selection, a significant number of patients will need salvage therapy. Adjuvant radiotherapy following radical prostatectomy is frequently used in patients with adverse features, such as pT3 stage, positive surgical margins or detectable PSA, after prostatectomy [2,3,4]. The combination of prostatectomy plus radiation has a negative functional effect both on erection and on continence recovery [5].

Adjuvant radiotherapy may cause significant urinary and rectal toxicity, especially if high-dose radiation is used [6]. Acute inflammatory disorders tend to be transitory, but late adverse effects tend to be more serious and include urinary incontinence caused by fibrotic contracted bladder and/or sphincteric damage [7]. The urethra, rhabdosphincter and or bladder neck may suffer alterations leading to impaired tissue elasticity, stricture formation and fibrotic changes with reduction of urethral mobility and consequent damage upon urinary continence. A prospective study confirmed that adjuvant external radiation after radical prostatectomy at a dose of 60 Gy resulted in an additional 6% of cases with urinary incontinence compared to prostate cancer under surveillance [8]. Furthermore, prostate cancer surgery or transurethral resection of the prostate (TURP) after radiation increased the risk of incontinence up to five-fold [9,10].

Surgical procedures to correct persistent stress urinary incontinence after prostate cancer surgery can be more challenging when performed on irradiated tissues than on naïve ones [11]. There is also a general belief that previous irradiation compromises the effectiveness of different incontinence devices, including male slings and an artificial urinary sphincter (AUS) [12,13,14].

The master trial showed that AUS and slings result in similar rates of incontinence [15]. Although only fixed slings were used as a comparator in the trial, adjustable devices like the adjustable trans-obturator male system (ATOMS) are increasingly used for the surgical correction of moderate-to-severe incontinence [16,17]. The purpose of the present study was to evaluate the effectiveness and safety of ATOMS (A.M.I. GmbH, Feldkirch, Austria) in patients with prostate cancer treated with both radical prostatectomy and radiotherapy. We also aimed to describe factors that identify the population with better outcomes in this presumably unfavorable scenario, complication rate and self-perceived satisfaction with the silicone-covered scrotal port (SSP) ATOMS.

## 2. Materials and Methods

### 2.1. Study Population

A retrospective multicenter study was undertaken to evaluate the effectiveness, safety and self-reported satisfaction in patients with stress incontinence after combined radical prostatectomy and radiotherapy intervened with SSP-ATOMS between 2016 and 2021 in eleven university hospitals from Europe and Canada. The study was approved by the Institutional Review Board and was conducted in accordance with the Helsinki Declaration. All patients included had persistent bothersome SUI for more than a year after radical prostatectomy refractory to pelvic floor exercises. In all cases, pelvic radiotherapy and radical prostatectomy were performed before ATOMS implant and minimum follow-up after ATOMS implant was 3 months.

Bladder neck contracture was not an exclusion criterion but stable urethral patency with a 17 Ch cystoscope was required. Additional local treatments of prostate cancer, severity of incontinence and patient age were not limiting factors for inclusion. The study was derived from current clinical practice. The indication for ATOMS was made by physician with the informed consent of the patient in every case.

The surgical technique for ATOMS placement followed the original description [18,19]. A 14 Ch Foley catheter was placed and delicate urethral dissection without splitting bulbospongiosus muscle was performed. Careful hemostasis was always performed, and drainage was not placed. When necessary, postoperative adjustment was performed in the office starting 2–3 weeks after the implantation by percutaneous injection of physiological sodium chloride solution through the SSP membrane and thereafter when required at intervals of 4 weeks until either dryness was achieved or maximum filling capacity of the system was reached.

### 2.2. Study Endpoints

The effectiveness and safety of the SSP-ATOMS in patients with stress incontinence after prostatectomy and adjuvant pelvic radiotherapy was evaluated. The primary endpoint was the dry patient rate, defined as no urine leakage or use of a single security pad with a pad-test ≤20 mL/day. The secondary endpoints were the complication rate defined as the Clavien–Dindo classification within the first three months after surgery [20], device removal rate and self-perceived satisfaction using the Patient Global Impression of Improvement (PGI-I) scale [21].

### 2.3. Variables Evaluated

The data analyzed included the patient age at the time of ATOMS implant, time of prostatectomy, adjuvant radiation of salvage prostatectomy, additional local treatments before prostatectomy (TUR-P) or before radiotherapy (brachytherapy), former history of bladder neck stricture, intraoperative and postoperative complications, continence outcomes and self-reported satisfaction with the procedure. The patients with a 24 h pad-test ≤20 mL/day were considered dry. Continence was evaluated baseline before ATOMS placement and postoperatively once device adjustment was considered complete. Incontinence severity was defined according to 24 h pads, also grouped regarding the number of pads/day (PPD) as mild (1–2 PPD), moderate (3–5 PPD) and severe (6 or more PPD).

The number and severity of complications was registered according to Clavien–Dindo classification within the first 3 months after the implant. Major complications were Grade III or beyond. Late complications including device surgical revision and device removal during follow-up were also evaluated. The indication of the device explant was exclusively based on the clinical decision. Pain was evaluated at discharge when available by the patient as a visual analogue scale (0 to 10). The self-assessed PGI-I scale was registered also when available, specified as the follow: 1, “very much better than before”; 2, “much better”; 3, “slightly better”; 4, “same”; 5, “worse”; 6, “much worse”; and 7, “very much worse”. For comparison, the results were pooled as 1–3 (at least better than before) vs. the rest.

### 2.4. Statistical Analysis

The statistics were calculated as the median values, interquartile range (IQR), minimum and maximum for continuous variables, and as the frequency and percent for the categorical data. The differences were calculated using the Wilcoxon test for continuous variables and the Fisher exact test for the categorical variables. A *p*-value < 0.05 was considered significant. A logistic regression was performed using a stepwise model (entry 0.15 and stay criterium 0.1) to evaluate the independent variables’ determinant of dryness after device adjustment. The area under the receiver operating characteristic (ROC) curve for the selected model and different combinations of predictive factors was calculated. The statistical analysis was developed using the Statistical Analysis System 9.3 (SAS Institute Inc., Cary, NY, USA).

## 3. Results

Two hundred and twenty-three consecutive patients with primary ATOMS implant for male stress incontinence after the combined treatment of prostate cancer with radical prostatectomy and adjuvant radiation were included in the study. Table 1 summarizes the clinical data. The median follow-up after the ATOMS implant was 30 (IQR: 23, range: 3–96) months.

### 3.1. Continence and Satisfaction Outcomes According to Incontinence Severity Baseline

Table 2 reveals the postoperative continence and satisfaction data for the total series analyzed and also after stratification according to incontinence severity. The mean change, both in PPD and in the pad-test, is expressed for the total series and for each incontinence severity group. The proportion of dry patients is inversely related to the severity of the baseline incontinence (*p* < 0.0001). On the other hand, the change in PPD and in the daily pad-test increased according to the severity of the baseline incontinence (each, *p* < 0.0001) and also in the number of postoperative adjustments needed (*p* < 0.0001). Noticeably, there was not an association between baseline incontinence and self-reported satisfaction (*p* = 0.67), possibly because the proportion of patients that rated their results as better than before was very high (84% in the total series with the self-reported satisfaction data).

### 3.2. Comparison of Effectiveness between Baseline and after Adjustment Data

**Figure 1:**
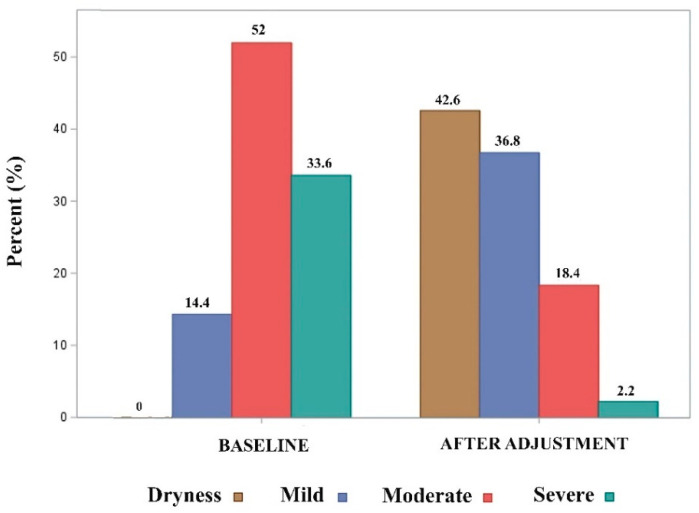
Evolution of the severity of incontinence according to the 24 h pad count before ATOMS implant and after adjustment.

Regarding our primary outcome, 42.6% of the patients included in the study (95 of 223 cases) achieved dryness. A comparative distribution of the incontinence severity before an ATOMS implant and adjustment revealed that 36.8% of the patients (82 cases) used 1–2 pads after adjustment, 18.4% (41 cases) used 3–5 PPD and only 2.2% (5 cases) used ≥6 PPD (compared to baseline; Bowker symmetry test, *p* < 0.0001). Globally, 20.6% of the patients remain with moderate or severe incontinence after adjustment (Figure 1).

**Figure 2:**
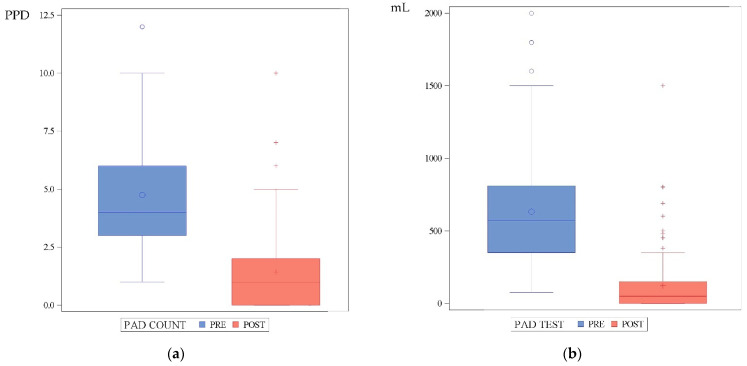
(**a**) Evolution of the 24 h pad-count (PPD); (**b**) evolution of the 24 h pad-test (mL).

Mean 24 h pad-count changed from 4.8 ± 2.3 (range: 1–12) PPD baseline to 1.4 ± 1.6 (range: 0–10) after adjustment (signed-rank test, *p* < 0.0001). Mean 24 h pad-test changed from 630 ± 386 (range: 75–2000) mL baseline to 120 ± 203 (range: 0–1500) mL after adjustment (signed-rank test, *p* < 0.0001) (Figure 2).

### 3.3. Postoperative and Late Complications

Intraoperative complications occurred in two patients (0.9%), both severe intraoperative bleeding. The mean visual analogue scale for pain (from 0 to 10) was evaluated in 102 patients (45.7%) at the time of hospital discharge, with a mean ± SD of 2.2 ± 1.8 (range: 0–7).

Postoperative complications within 90 days after surgery presented in 36 (16.1%) cases, one with two different complications. According to the Clavien–Dindo system, 20 (9%) were grade I, 11 (4.9%) grade II and 5 (2.2%) grade III. In order of frequency, 37 complications were pain in 14 (6.3%); urinary retention in 8 (3.6%); scrotal hematoma and wound infection in 3 each (1.4%); device infection, wound dehiscence and paresthesia in 2 each (0.9%); and urgency, gout and port displacement needing reposition in 1 each (0.4%).

At a mean on 36 ± 21 (range: 3–86) months of follow-up, surgical revision was performed in 29 cases (13%) and the device was explanted in 26 (11.7%). The reasons for revision were persistent incontinence in 10 (4.5%); scrotal port skin erosion and pain in 6 each (2.7%); device infection in 4 (1.8%); port displacement needing reposition in 2 (0.9%); and wound dehiscence in 1 (0.5%).

### 3.4. Self-Perceived Satisfaction

**Figure 3:**
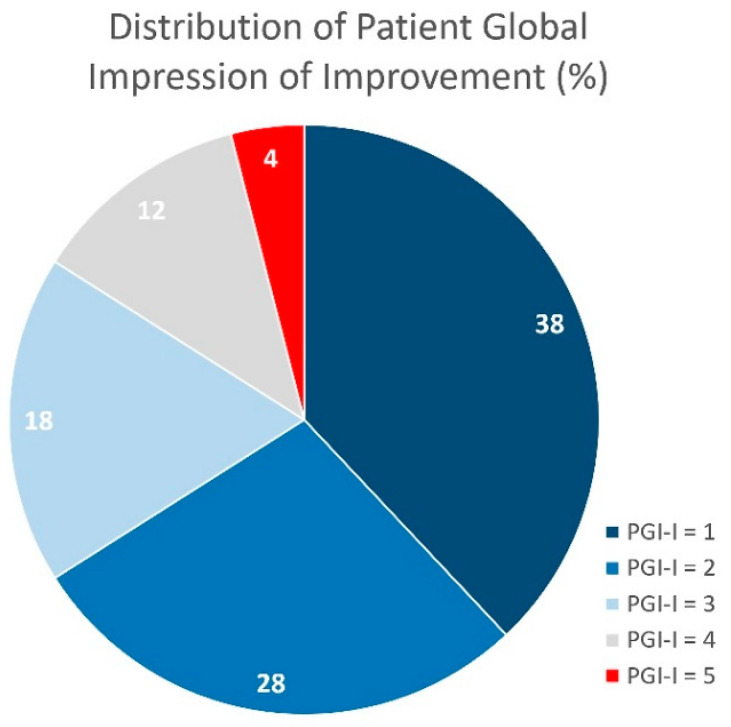
Percent of each PGI-I score with ATOMS (*n* = 125; 1, very much better; 2, much better; 3, slightly better; 4, same; 5, worse).

Postoperative self-perceived satisfaction using the Patient Global Impression of Improvement (PGI-I) scale was registered in 125 patients (56%). The mean PGI-I score after adjustment compared to before the ATOMS was 2.2 ± 1.2 (range: 1–5). The proportion of patients with each PGI-I score is represented in Figure 3. Patients with at least a better perception than before (PGI-I from 1 to 3) was registered in 84% of the cases evaluated with this tool.

### 3.5. Logistic Regression Analysis for Factors Predictive of Dryness

A univariate analysis of factors associated with dryness (no leakage or one security PPD with ≤20 mL) after an ATOMS adjustment were patient age at the time of implant (unfavorable for older age; *p* = 0.06), the sequence between prostatectomy and radiation (unfavorable for salvage prostatectomy; *p* = 0.08), no previous incontinence surgery (*p* = 0.02), absence of overactive bladder symptoms baseline (*p* = 0.04), absence of bladder neck stricture (*p* = 0.001), no need of surgical revision (*p* = 0.008) and lower baseline incontinence severity (*p* = 0.0003) (Table 3).

A multivariate analysis identified that an absence of surgical revision (*p* = 0.018), absence of bladder neck stricture (*p* = 0.05), primary prostatectomy (*p* = 0.07) and baseline incontinence severity (*p* < 0.0001) were independent predictors of dryness (Table 3).

**Figure 4:**
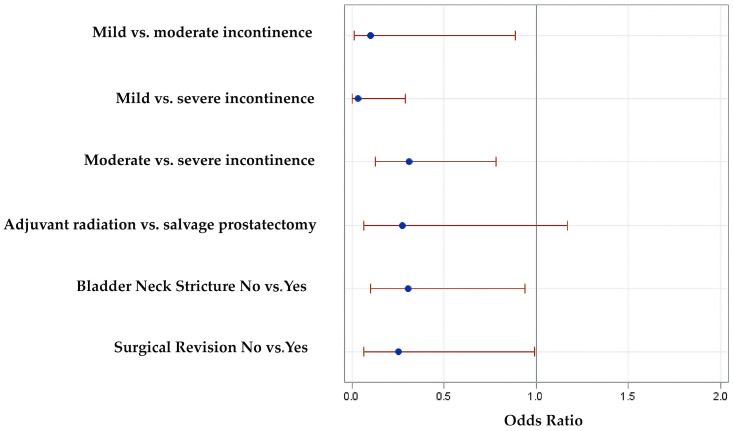
Odds ratios with 95% confidence limits for the independent variables identified.

A logistic regression model for the prediction of ATOMS success (i.e., 24 h pad-test ≥20 mL after ATOMS adjustment) in this population was built (Figure 4). The area under the curve for this predictive model was 79%. The model was internally validated by bootstrapping with 74.5 (95% CI 74.2–74.8)% apparent performance and 9.4 (95% CI 9.2–9.5)% expected optimism.

### 3.6. Nomogram to Predict Dryness after ATOMS Adjustment

**Figure 5:**
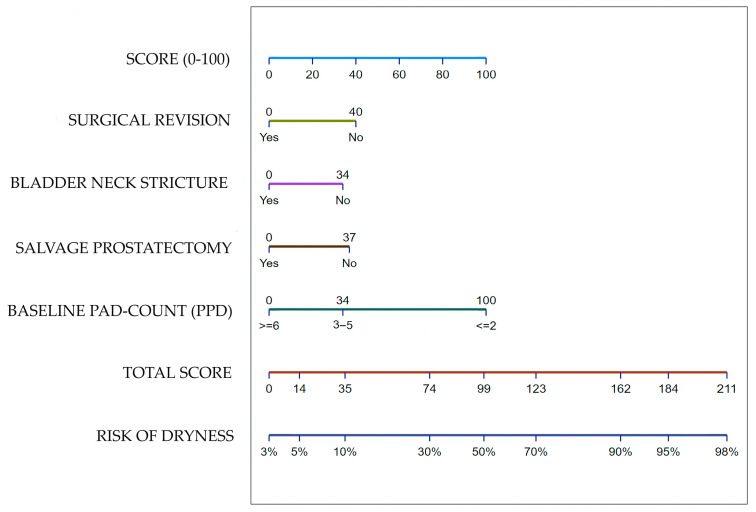
Nomogram to predict dryness with ATOMS after prostatectomy and radiation.

A nomogram to facilitate the prediction of dryness in patients treated with ATOMS after radical prostatectomy and radiation is proposed (Figure 5).

## 4. Discussion

The impact of the association of pelvic radiotherapy with radical prostatectomy on male continence after prostate cancer treatment is tremendous. According to data from the National Health and Nutrition Examination Surveys (NHANES), incontinence presented in 23% of men who underwent radical prostatectomy, 12% treated with radiotherapy and 52% of men treated with the combination of them [22].

On the one hand, when first local treatment is prostatectomy, the use of adjuvant or salvage radiotherapy severely affects continence recovery after surgery, but the toxicity profile of immediate postoperative radiation or delayed salvage radiotherapy could be different. Clinicians are prone to postpone the use of radiotherapy to maximize continence recovery, but this could have an impact on therapeutic reduced efficacy [23,24]. The optimal radiation dose delivered after prostatectomy and fractionation could also affect continence recovery [25]. On the other hand, salvage prostatectomy after failed radiation can provide local control of disease but with 25–79% of patients being incontinent [26].

Initially, a very interesting success rate was reported with the retrourethral transobturator fixed sling in selected patients after radical prostatectomy and adjuvant radiotherapy [27]. However, soon caution was later advised in recommending a fixed sling in patients with a history of pelvic irradiation [12]. More recently, severely compromised long-term functional outcomes and patient satisfaction were confirmed [28,29]. The Virtue male quadratic sling is another interesting option, as it provides bidirectional compression and elevation of the bulbous [30]; however, there is no information regarding its use in patients with previous radiation. A recent systematic review and meta-analysis concluded that adjustable slings might lead to higher objective cure rates than fixed ones, but randomized controlled trials with long-term follow-up and the same definition for continence are needed [31].

Several multicenter studies identified pelvic irradiation is a factor that negatively affects the results of ATOMS [32,33,34,35,36]. Also, a systematic review and meta-analysis confirmed that the proportion of irradiated patients included in the different studies available affected reported dryness rate, thus providing a source of heterogeneity [37]. In as much, a prospective observational study confirmed that the main factors that predict development of postoperative complications after ATOMS implant were previous radiotherapy and surgery for urethral stricture [38]. However, radiation does not compromise the high self-reported satisfaction with the ATOMS [39]. Therefore, although expected outcomes with ATOMS after radiation can be compromised, the device is still an option for the correction of male stress incontinence in patients with prostate cancer treated with prostatectomy and adjuvant radiation.

This belief is partly supported by the observation that in no case reported to date the ventral compression of the ATOMS has produced urethral erosion or urethral atrophy, complications that typically occur after the circumferential periurethral placement of AUS [40]. This observation makes an ATOMS device especially attractive for patients with a fragile urethra due to a previously failed AUS or failed retrobulbar sling [41,42] and also for patients previously treated for urethral stricture or bladder neck contracture [43], provided of course that some residual sphincter function remains. Conversely, the risk of urethral erosion with increased surgical revision and device explant was confirmed in several studies on patients implanted with an AUS after radiotherapy [44,45]. Radiation and prior urethroplasty were also confirmed independent risk factors for earlier time to erosion with an AUS [46]. Mainly for this reason, the role and outcomes of AUS after pelvic irradiation remain controversial. Some series did not evidence different outcomes [47,48,49], although other studies confirmed a higher risk of surgical revision and device explant [44,45]. Finally, several multicenter studies confirmed that pelvic irradiation adversely affects AUS survival for increased and earlier urethral erosion leading to device explant [11,50].

Radiation causes a variety of tissue alterations that include vascular changes, fibrosis, cellular depletions and inflammation [51]. Histopathological examination of radiated tissue demonstrated vascular loss and increased scarring in the membranous urethra and in the bladder neck, changes that may also facilitate stricture formation, thus causing confusion of the deleterious factors involved in continence status [52]. In fact, we have confirmed that in radiated patients, bladder neck stricture is a negative predictor of ATOMS results. Similarly, when evaluating the role of ATOMS in patients with previously treated urethral stricture or bladder neck contracture, we identified that radiation was also a confounding factor [43]. Despite the high risk of problems in the irradiated male patients with urethral fragility, the AUS still remains the most frequently used option; however, the ATOMS could be an alternative to consider, especially in patients with failed previous AUS and residual sphincteric function [41,42]. In fact, recent analysis confirmed the long-term durability in the efficacy of ATOMS after radiation with 62.5% social continence after 5 years mean follow-up [53].

Overactive bladder (OAB) symptoms in irradiated patients may also be a crucial issue to evaluate postoperative efficacy of the device. There are no data to suggest that ATOMS contributes itself to development of de novo OAB; however, that did present in 5% of the cases in this series. In fact, a positive correlation between previous radiotherapy and postoperative OAB was proposed [54]. In the univariate analysis we performed, a lack of OAB symptoms were associated to dryness in the population evaluated, but this was not an independent factor in the multivariate analysis. The same happened with the absence of bladder neck stricture and older patient age.

We confirmed that the timing between radiotherapy and radical prostatectomy has an independent impact on the results of ATOMS, with worse efficacy in patients treated first with radiation and later with salvage prostatectomy. This is a relatively infrequent situation nowadays, as the favored combination of treatments is early salvage treatment would because it offers the opportunity to spare many men radiotherapy and its associated side effects [4,5]. However, because of the characteristics of the database we used, we were not able to address very important items regarding the mode of radiation, including the time between surgery and radiation, total dose and fractionation. All these factors contribute to the prediction of continence recovery [25,26,55] and could also have an influence on the success rate of ATOMS in these patients, and we are sorry to recognize that they remain unstudied. Similarly, the approach used for radical prostatectomy is another missing variable, despite there being no previous report to alert maybe having an influence on the results of the ATOMS implant. Other variables that have not been evaluated include body mass index, ASA score, Charlson comorbidity, prostate cancer risk and operative findings. The evolution of pain after hospital discharge has not been registered for this study either.

Also, the study is limited by the absence of a comparison group. In fact, we could not answer the really important question of whether combined prostatectomy and radiation implies worse results after an ATOMS implant and adjustment, compared to the outcomes of patients treated with radical prostatectomy alone. A study based on a larger population of patients and using propensity score matching to compare groups will serve to settle the enigma of whether an ATOMS performs equally or worse in patients with or without radiation. In this sense, we do not know either whether the security profile is impacted. The description of complications, revision rate and explant rate we evidenced is totally in line with that of the general population of patients with ATOMS at a similar mean follow-up [34,38,56]. Also, the reasons for surgical revision we detected in this series is very much like those in the general population [40]. As far as we know, there is no other publication centering on the description of results with ATOMS in patients with adjuvant radiation exclusively. Despite the limitations acknowledged, the findings presented are encouraging to further analyze the issue in multicenter studies with a larger number of patients and better control of confounding variables.

In summary, total continence was achieved in 42.6% of the patients treated with adjuvant radiotherapy (65.6% for mild incontinence, 48.3% for moderate and 24% for severe) and 84% of them self-reported as satisfied compared to their situation baseline. The logistic regression analysis we performed served to identify the variables necessary to foresee the effectiveness of ATOMS in this population. Baseline incontinence severity considering the pad-test is the most determinant variable, with patients leaking up to 5 PPS (mild and moderate incontinence) performing better. The second factor limiting efficacy is the need for surgical revision during follow-up. Pain, scrotal skin erosion and device infection are complications that may lead to surgical revision, performed in 13% of the cases at a mean 3 years follow-up. Additional predictive factors at the limit of statistical significance are bladder neck stricture and salvage prostatectomy performed after failed radiation. The nomogram built for the prediction of dryness can be a useful tool to counsel a patient in the decision to receive an ATOMS implant after prostatectomy and radiotherapy.

## 5. Conclusions

ATOMS can be used to correct or improve male stress incontinence in patients with a history of prostate cancer treated with the combination of prostatectomy and radiotherapy. The efficacy and safety data with this approach are in consonance with the data reported from the general population and mainly depend on the degree of baseline incontinence. Patient satisfaction with the device is also high, regardless of the baseline incontinence severity. ATOMS is a very interesting option for reconstructive and functional urologists facing complex cases of male incontinence. Our experience can be useful to counsel patients with prostate cancer treated with surgery plus radiation.

## Figures and Tables

**Table 1:** Preoperative, operative and postoperative data of patients included in the study.

Variable	Total Series (*n* = 223)
Preoperative data	
Age, years, median (IQR)	71 (11)
Time since prostatectomy to ATOMS, months, median (IQR)	52 (58)
Previous incontinence surgery, *n* (%)	27 (12.1)
Salvage prostatectomy, *n* (%)	12 (5.4)
Previous TUR-P, *n* (%)	8 (3.6)
Previous brachytherapy, *n* (%)	2 (0.9)
Androgen deprivation therapy, *n* (%)	46 (20.6)
Bladder neck stricture, *n* (%)	63 (28.2)
OAB symptoms, *n* (%)	27 (12.1)
24 h pad-count, PPD, median (IQR)	4 (3)
24 h pad-test, mL, median (IQR) ^1^	570 (460)
Operative data	
Operative time, min., median (IQR)	62 (24)
Intraoperative complication, *n* (%)	2 (0.9)
VAS for pain (0–10), median (IQR) ^2^	2 (2)
Postoperative data	
Postoperative complications ^3^, any grade, *n* (%)	36 (16.1)
Grade I ^3^, *n* (%)	20 (9)
Grade II ^3^, *n* (%)	11 (4.9)
Grade III ^3^, *n* (%)	5 (2.2)
Surgical revision, *n* (%)	29 (13)
Device explant, *n* (%)	26 (11.7)
De novo OAB symptoms, *n* (%)	11 (4.9)
Total filling volume, mL, median (IQR)	16 (11.5)
Number of fillings, median (IQR)	1 (3)
Patients with pad-test ≤20 mL, *n* (%)	95 (42.6)
24 h pad-count, PPD, median (IQR)	1 (2)
24 h pad-test, mL, median (IQR) ^4^	50 (150)
PGI-I = 1 (very much better), *n* (%) ^4^	48 (38.4)
PGI-I = 2 (much better), *n* (%) ^4^	35 (28)
PGI-I = 3 (slightly better), *n* (%) ^4^	22 (17.6)
PGI-I = 4 (same), *n* (%) ^4^	15 (12)
PGI-I = 5 (worse), *n* (%) ^4^	5 (5)

^1^ Baseline pad-test evaluated in 125 patients. ^2^ Pain evaluated in 102 patients at hospital discharge, usually on day 1 after surgery. ^3^ According to Clavien–Dindo classification. ^4^ PGI-I evaluated in 125 patients. IQR, Interquartile range; PPD, pads-per-day; VAS, Visual Analogue Scale; OAB, overactive bladder; PGI-I, Patient Global Impression of Improvement.

**Table 2:** Outcomes and mean change in postoperative incontinence for each incontinence severity group after ATOMS implant in patients treated with radical prostatectomy and adjunct radiation.

Degree of Incontinence	N (%)	Dryness * N (%)	Satisfaction ^#^ N (%)	Mean Change in PPD	Mean Change in Pad-Test ^@^	Mean Number of Adjustments
Total series	223 (100)	95 (42.6)	105 (84)	3 ± 2.5	450 ± 400	3 ± 3
Mild (1–2 PPD)	32 (14.3)	21 (65.6)	10 (90.9)	1.5 ± 1	200 ± 130	2.5 ± 3.5
Moderate (3–5 PPD)	116 (52)	56 (48.3)	52 (85.3)	3 ± 2	350 ± 200	3 ± 3
Severe (≥6 PPD)	75 (33.7)	18 (24)	43 (81.1)	5 ± 2	700 ± 270	4 ± 2
*p*-Value		<0.0001	0.67	<0.0001	<0.0001	<0.0001

* 24 h Pad-test < 20 mL. ^#^ PGI-I 1–3, evaluated in 125 patients. PPD, pads/day. ^@^ Pad-test evaluated in 125 patients.

**Table 3:** Analysis of factors involved in the prediction of dryness.

**Univariate Analysis**	**OR**	**95% CI Upper Limit**	**95% CI Lower Limit**	***p*-Value**
Patient age <70 vs. ≥70 years	0.59	0.34	1.01	0.06
Adjuvant radiotherapy vs. salvage prostatectomy	0.35	0.1	1.2	0.08
Primary incontinence surgery	0.34	0.13	0.89	0.02
OAB symptoms	0.37	0.14	0.96	0.04
Bladder neck stricture	0.35	0.18	0.67	0.001
Surgical revision	0.32	0.13	0.77	0.008
Baseline incontinence: mild vs. moderate	0.54	0.23	1.25	0.0003
Mild vs. severe	0.21	0.09	0.50	
Moderate vs. severe	0.40	0.22	0.73	
**Multivariate Analysis**	**OR**	**95% CI Upper Limit**	**95% CI Lower Limit**	***p*-Value**
Surgical revision	0.25	0.06	0.99	0.018
Adjuvant radiotherapy vs. salvage prostatectomy	0.28	0.07	1.17	0.07
Bladder neck stricture	0.3	0.1	0.94	0.05
Baseline incontinence: mild vs. moderate	0.1	0.01	0.89	<0.0001
Mild vs. severe	0.03	0	0.29	
Moderate vs. severe	0.31	0.13	0.78	

CI: confidence interval; OAB: overactive bladder.

## Data Availability

Full data will be available upon reasonable request to the corresponding author.
